# Evaluation of the Role of *Candida albicans* Agglutinin-Like Sequence (*Als*) Proteins in Human Oral Epithelial Cell Interactions

**DOI:** 10.1371/journal.pone.0033362

**Published:** 2012-03-12

**Authors:** Celia Murciano, David L. Moyes, Manohursingh Runglall, Priscila Tobouti, Ayesha Islam, Lois L. Hoyer, Julian R. Naglik

**Affiliations:** 1 Department of Oral Immunology, King's College London Dental Institute, King's College London, London, United Kingdom; 2 Department of Pathobiology, University of Illinois at Urbana-Champaign, Urbana, Illinois, United States of America; New Jersey Medical School - University of Medicine and Dentistry of New Jersey, United States of America

## Abstract

The fungus *C. albicans* uses adhesins to interact with human epithelial surfaces in the processes of colonization and pathogenesis. The *C. albicans ALS* (agglutinin-like sequence) gene family encodes eight large cell-surface glycoproteins (Als1-Als7 and Als9) that have adhesive function. This study utilized *C. albicans* Δ*als* mutant strains to investigate the role of the Als family in oral epithelial cell adhesion and damage, cytokine induction and activation of a MAPK-based (MKP1/c-Fos) signaling pathway that discriminates between yeast and hyphae. Of the eight Δ*als* mutants tested, only the Δ*als3* strain showed significant reductions in oral epithelial cell adhesion and damage, and cytokine production. High fungal:epithelial cell multiplicities of infection were able to rescue the cell damage and cytokine production phenotypes, demonstrating the importance of fungal burden in mucosal infections. Despite its adhesion, damage and cytokine induction phenotypes, the Δ*als3* strain induced MKP1 phosphorylation and c-Fos production to a similar extent as control cells. Our data demonstrate that Als3 is involved directly in epithelial adhesion but indirectly in cell damage and cytokine induction, and is not the factor targeted by oral epithelial cells to discriminate between the yeast and hyphal form of *C. albicans*.

## Introduction

*C. albicans* is a commensal fungus of the human oro-gastrointestinal and vaginal tracts [1], [2]. When the immune system is compromised, or when the normal microbiota are disrupted, debilitating and often recurring mucosal diseases can result. *C. albicans*, the most common clinical *Candida* isolate, uses adhesins, hypha formation, phenotypic switching and production of extracellular hydrolytic enzymes to interact with its human host [3]–[6]. Among *C. albicans* adhesins is the Als (agglutinin-like sequence) family, encoded by eight distinct genetic loci (*ALS1* to *ALS7*, *ALS9*; [7], [8]). Als proteins have a similar structure, including an N-terminal secretory signal sequence, followed by an NT domain of approximately 320 amino acids, a T domain of approximately 104 amino acids, a TR domain of head-to-tail copies of a Ser/Thr-rich repeated sequence, and a Ser/Thr-rich C domain of variable size and sequence. Mature Als molecules are large glycoproteins that are linked to β-1,6 glucan in the *C. albicans* cell wall [9]. For example, the estimated sizes for mature Als1 and Als3 are 600 and 440 kDa, respectively.

Because of its proposed similarity to functional domains of other adhesion proteins, the Als NT domain is often studied in the absence of the remainder of the mature molecule. X-ray crystallography and NMR were used to solve the structure of the Als9-2 (amino acids 18–328) and Als1 (amino acids 18–329) respectively [10]. This work showed highly similar structures for the two proteins that feature two tandem Dev-IgG type immunoglobulin domains and a cavity, with an invariant Lys residue, which binds to the C-terminal carboxyl group of peptides with broad binding specificity. It is very likely that these structural features will be found in the corresponding fragments from the remaining proteins in the Als family, although the other structures must still be solved. Als proteins, particularly Als3, contribute to biofilm formation, mediate epithelial invasion and induce epithelial cell damage [11]. Als3 has been the focus of considerable investigation since it is produced so abundantly on the surface of germ tubes and hyphae [12], providing a potential intersection between adhesive function and hypha formation. Hypha formation is also very important in mucosal pathogenicity [13]–[15].

As part of our studies of interactions between *C. albicans* and oral epithelial cells, we discovered a mechanism that enables oral epithelial cells to discriminate between *C. albicans* yeast and hyphae via a mitogen-activated protein kinase (MAPK) signaling pathway [16]–[19]. This discriminatory mechanism targets *C. albicans* hyphae and constitutes activation of the MAPK phosphatase MKP1 and c-Fos transcription factor, which are involved in the induction and regulation of a proinflammatory cytokine response. Since the *ALS* gene family is expanded in *C. albicans* and has adhesion/invasion functions, we sought to determine the role of this family in epithelial adhesion and induction of cell damage. Furthermore, given that Als3 is abundant on hyphae, we wanted to determine whether Als3 is the moiety that mediates activation of the MAPK-based MKP1/c-Fos signaling pathway leading to cytokine induction.

## Materials and Methods

### Growth of *C. albicans* strains and the epithelial cell line

*C. albicans* strains used in this work included the wild-type strain SC5314 [20] and strains in which both alleles of an individual *ALS* gene were deleted. The collection of mutants included 1467 (Δ*als1*; [21]), 2342 (Δ*als2/P_Mal_-ALS2*; [22]), 1843 (Δ*als3*; [21]), 2034 (Δ*als4*; [22]), 2373 (Δ*als5*; [23]), 1420 (Δ*als6*; [23]), 1429 (Δ*als7*; [23]) and 2028 (Δ*als9*; [24]). Strain CAI4 [25], a Ura-negative derivative of SC5314 was the parent for each of the mutant strains. CAI4 was transformed with plasmid CIp10 that encodes the *URA3* gene [26] for the purposes of creating a Ura3-positive control for this work. This strain is referred to as ‘CAI4’ throughout the manuscript. *C. albicans* strain 2322 was also used as a control [27]. This strain is the Δ*als3* mutant, into which a copy of the *ALS3* large allele from strain SC5314 was reintegrated into the *ALS3* locus. *C. albicans* were grown in YPD medium (1% yeast extract, 2% peptone, 2% dextrose) overnight at 30°C to saturation prior to experimentation. Experiments used a buccal epithelial squamous cell carcinoma line, TR146 [28]. TR146 monolayers were grown in Dulbecco's Modified Eagle's Medium (DMEM) supplemented with 10% fetal bovine serum (FBS). Prior to experiments, TR146 confluent monolayers were serum-starved overnight and serum-free DMEM used during the next day's experiments.

### Adherence assay and morphological analysis

TR146 oral epithelial cell monolayers were grown to confluence in six-well tissue culture plates and serum-starved overnight. 100, 200 or 300, depending on the assay, *C. albicans* yeast cells were added to replicate wells containing 1 ml serum-free DMEM for 90 min at 37°C and 5% CO_2_. *C. albicans* contact with epithelial cells is a strong inducer of hypha formation, and by 90 min significant germ tube formation has already occurred. Following incubation, non-adherent *C. albicans* cells were removed by aspiration. Each well was washed twice with 1 ml PBS, and overlaid with molten Sabouraud's dextrose agar (SDA) at 45°C. Plates were then incubated at 30°C for 24 h, and the resulting colonies counted. Replicate control plates of the initial inoculum were also incubated and the resulting colonies counted. Percent adherence was calculated as (mean adherent CFU/mean total CFU)×100. The experiments were repeated on two separate occasions. For morphological analysis, CAI4 and Δ*als3* strains were inoculated onto TR146 monolayer cultures at a MOI of 10. After 90 min monolayers were fixed in 10% buffered formalin and examined by differential interference contrast (DIC) microscopy at ×400.

### Cell damage assay

TR146 confluent monolayers were grown in 24-well tissue culture plates. *C. albicans* yeast cells were added to TR146 monolayers and incubated at 37°C and 5% CO_2_ for 24 h. A fungal:epithelial cell multiplicity of infection (MOI) between 0.01 and 10 was used. Control wells contained PBS alone. Following incubation, culture supernatant was collected and assayed for lactate dehydrogenase (LDH) using the Cytox 96 Non-Radioactive Cytotoxicity Assay kit (Promega) according to manufacturer's instructions. A recombinant porcine LDH (Sigma-Aldrich) was used to generate a standard curve and sample values extrapolated from the curve. The assay was conducted using a single well on three separate occasions. Replicate LDH measurements were made on the single well. Data were analyzed using a two-tailed t-test with p<0.05 considered significant.

### Measurement of cytokine levels

*C. albicans* cells were added to TR146 monolayers, grown in 24-well tissue culture plates, and incubated as described above. At 24 h, culture supernatants were used to measure cytokine levels (IL-1α, IL-6, G-CSF, GM-CSF) using the Fluorokine MAP cytokine multiplex microbead assay kits (R&D Systems), coupled with the Bioplex 200™ machine according to the manufacturer's protocol. The assay was conducted using a single well on three separate occasions. Replicate cytokine measurements were made on the single well. Data were analyzed using a two-tailed t-test with p<0.05 considered significant.

### Western blotting

Western blotting was used to assess phosphorylation of MKP1 and induction of c-Fos in TR146 monolayers that were incubated with *C. albicans*. *C. albicans* yeast cells were added to the TR146 monolayers, grown in 12-well tissue culture plates, and incubated for 2 h at 37°C and 5% CO_2_. Epithelial cells were lysed using a modified RIPA lysis buffer (50 mM Tris-HCl (pH 7.4), 150 mM NaCl, 1 mM EDTA, 1% Triton X-100, 1% sodium deoxycholate, 0.1% SDS) containing inhibitors of protease (Perbio, UK) and phosphatase (Sigma-Aldrich, UK). The lysate was incubated on ice for 30 min and centrifuged for 10 min in a refrigerated microfuge. Supernatants were assayed for total protein using the BCA Protein Quantitation kit (Perbio, UK). Protein (15 µg) was separated on a 12% NuPAGE Bis:Tris minigel (Invitrogen, UK) before transfer to a PVDF membrane (GE Healthcare). α-actin was used as a loading control. Membranes were incubated with primary (1∶1000 dilution) and secondary (1∶10,000 dilution) antibodies and developed using Immobilon chemiluminescent substrate (Millipore, UK). Rabbit monoclonal antibodies to human phospho-MKP1 and c-Fos were purchased from Cell Signaling Technologies (New England Biolabs, UK). Mouse monoclonal antibody to human α-actin was purchased from Millipore and goat anti-mouse and anti-rabbit horseradish peroxidase (HRP)-conjugated antibodies were from Jackson Immunologicals Ltd (Stratech Scientific, UK). The assay was conducted on four independent occasions, inoculating a single TR146 well with each *C. albicans* strain.

### Stimulation of epithelial monolayers with recombinant Als3

Soluble, purified NT-Als3 was prepared as described previously [12]. Briefly, a vector encoding the first 329 amino acids of Als3 and a hexa-His tail was transformed into *Pichia pastoris*. Soluble Als3 was secreted from the recombinant strain, producing a protein (amino acids 18 to 329) from which the secretory signal sequence was cleaved. The protein was purified by His-Trap column chromatography (GE Healthcare), dialyzed against phosphate buffered saline and its concentration determined using the Bradford method (Bio-Rad). The NT-Als3 protein was added to TR146 monolayers in the context of the cell damage, cytokine induction, MKP1 phosphorylation and c-Fos assays described above. Concentrations added ranged from 0.3–10 µg/ml. Assays were conducted on two separate occasions. Controls included PBS alone. Data analysis followed the methods described above.

## Results

### Δ*als3* has reduced adhesion and damage to oral epithelial cells

**Figure 1:**
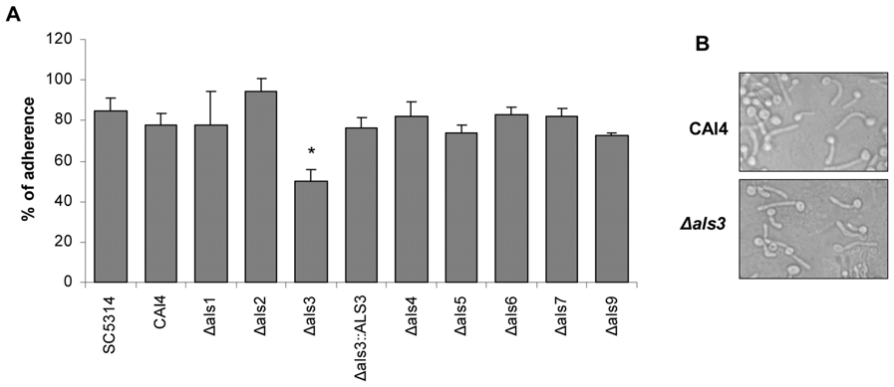
Adhesion of *C. albicans* Δ*als* mutants to TR146 oral epithelial cell monolayers. *C. albicans* yeast cells (100 cfu) were added to TR146 monolayers and incubated at 37°C for 90 min. Each Δ*als* strain was tested individually and compared to the wild-type strain SC5314 (*URA3/URA3*) and the CAI4 control (*ura3::URA3*). After extensive washing, molten (45°C) Sabouraud's dextrose agar was added and the plates were then incubated at 30°C for 24 h for colony development of adhered *C. albicans* cells. Results were expressed as the percentage of adhered *C. albicans* cells. Data represent mean values ± SEM and are representative of two independent experiments. * p<0.05 compared to CAI4. (A) % adherence of Δ*als* strains; only Δ*als3* showed significantly decreased adhesion to TR146 monolayers. (B) The parent strain CAI4 and the Δ*als3* strain were added to TR146 oral epithelial cells and incubated at 37°C for 90 min. The monolayers were then formalin fixed and morphology was assessed by DIC microscopy at ×400.

**Figure 2:**
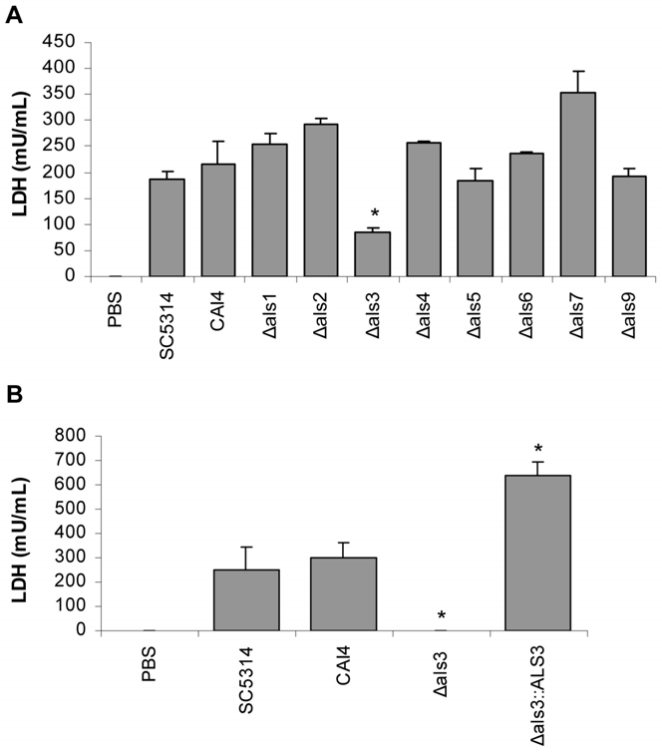
Induction of cell damage by *C. albicans* Δ*als* mutants. *C. albicans* yeasts were added to TR146 monolayers and incubated under standard conditions for 24 h. A fungal:epithelial cell MOI of 0.01 was used. Cell culture medium was collected and assessed for lactate dehydrogenase (LDH) release as a measure of epithelial cell damage induced by *C. albicans* Δ*als* mutants (A) or the *ALS3* reintegrant strain (B). Data represent mean values ± SEM and are representative of three independent experiments. * p<0.05 compared to CAI4. The Δ*als3* strain was the only one that showed significantly reduced cell damage compared to the control. Reintegration of a wild-type *ALS3* allele restored cell damage capability to the strain.

The TR146 oral epithelial carcinoma cell line is one of the most common cell lines currently used to investigate *C. albicans*-oral epithelial cell interactions [13], [14], [16]–[18], [29], [30]. We tested each of the Δ*als* mutants for its ability to adhere to TR146 cells after 90 min of co-incubation. Only the Δ*als3* mutant was defective in adhesion in this assay, with approximately 30–40% reduced adhesion compared to control strains (Fig. 1a). Notably, the percentage of Δ*als3* cells forming germ tubes and the morphology of the germ tubes could not be distinguished from the wild-type strain (Fig. 1b). The Δ*als3* strain was also the only one of the set of **Δ** *als* mutants to show significantly decreased damage to TR146 cells following 24 h of incubation (Fig. 2a). Although damage to TR146 cells was reduced by nearly 75% for the Δ*als3* strain, a small amount of epithelial cell damage was still observed. Because adhesion is a prerequisite for invasion and subsequent cell damage, this result is consistent with the adhesion assay data. Notably, at 24 h, the Δ*als3* strain showed decreased adhesion to the TR146 monolayer compared with the control (data not shown).

Because the Δ*als3* mutant showed significant reductions in the adhesion and cell damage assays, the assays were repeated to include the control strain 2322, into which an *ALS3* allele was reintegrated. Restoration of wild-type adhesion (Fig. 1b) and cell damage (Fig. 2b) for this strain suggested that the results observed for the Δ*als3* mutant were attributable directly to loss of Als3. Variation between biological replicates was evident when comparing the various assays. For example, cell damage data for the Δ*als3* mutant varied between experiments (compare Fig. 2a and b); however, in both instances the trends were the same and damage caused by the Δ*als3* strain was reduced significantly compared to the control. It was also unclear why the *ALS3* reintegrant strain 2322 showed higher LDH levels than strains SC5314 and CAI4, however, the strain clearly demonstrated rescue of the cell damage phenotype.

### Reduced capacity of Δ*als3* to induce epithelial cytokines

**Figure 3:**
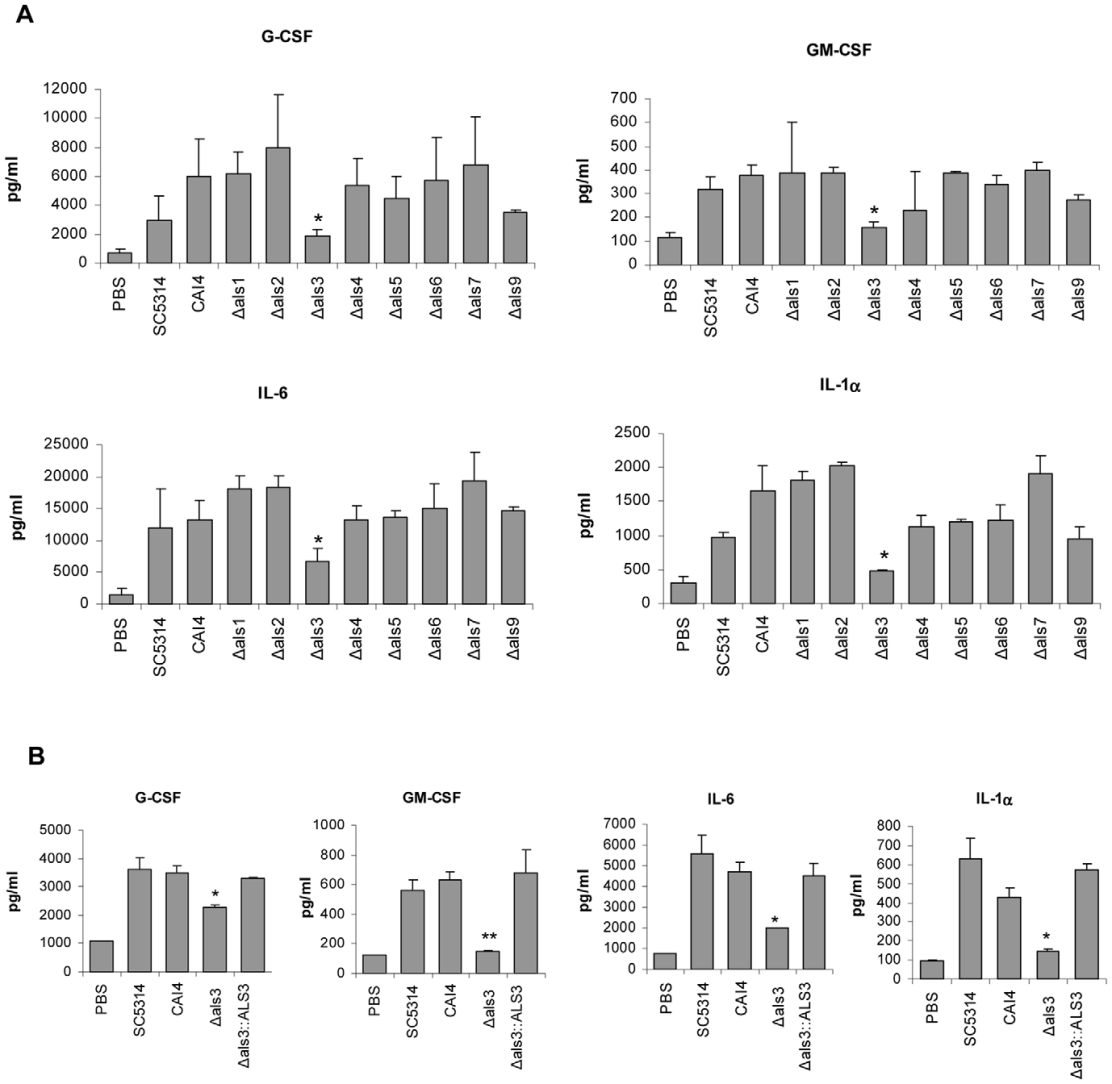
Cytokine induction by *C. albicans* Δ*als* mutants. *C. albicans* yeasts were added to TR146 epithelial cell monolayers and incubated under standard conditions for 24 h. A fungal:epithelial cell MOI of 0.01 was used. Cell culture medium was collected and cytokine levels measured using a multiplex microbead assay. Data represent mean values ± SEM and are representative of three independent experiments. * p<0.05 compared to CAI4. (A) The Δ*als3* strain was the only one that showed significantly reduced cytokine production compared to the control. Reintegration of a wild-type *ALS3* allele restored the native cytokine production to the strain (B).

One marker of epithelial activation in response to an invading pathogen is the induction of a pro-inflammatory cytokine response. The ability of Δ*als* mutants to induce cytokines from epithelial cells has not been investigated previously. Because of their role in adhesion to epithelial cells, one would expect that Als-epithelial receptor interactions at the cell surface might trigger cytokine production. We assessed whether Δ*als* mutants were able to induce cytokine production in TR146 cells 24 h following inoculation with *C. albicans* at a MOI of 0.01. In this assay, only the Δ*als3* mutant was deficient in inducing G-CSF, GM-CSF, IL-6 and IL-1α (Fig. 3a), which we previously demonstrated as being the main read-out cytokines induced by *C. albicans* for TR146 cells [16]. Repetition of the cytokine assays using the *ALS3* reintegrant control strain showed that the results observed for the Δ*als3* mutant were due to the loss of Als3 (Fig. 3b).

### Δ*als3* induces MKP1 phosphorylation and c-Fos production

**Figure 4:**
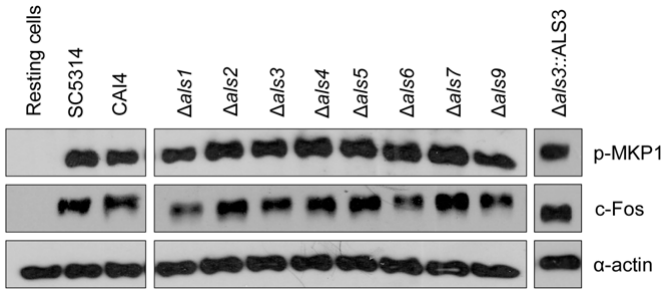
Activation of MKP1 and c-Fos signaling by *C. albicans* Δ*als* mutants. *C. albicans* yeasts were added to TR146 oral epithelial cell monolayers and incubated under standard conditions for 2 h. Results from a fungal:epithelial cell MOI of 10 are shown here. Epithelial cell lysates were separated by SDS-PAGE and Western blotted to detect MKP1, c-Fos or α-actin (positive control). Data are representative of four independent experiments. There was no difference in MKP1 or c-Fos between control *C. albicans* strains (SC5314 and CAI4) and the Δ*als* mutants.

Epithelial cells discriminate between the yeast and hyphal form of *C. albicans* via a MAPK-based mechanism that targets the hyphal form, which involves MKP1 and c-Fos activation [16]. Given that the Δ*als3* mutant forms normal germ tubes/hyphae in the epithelial cell culture conditions used in these experiments, but was deficient in adhesion (Fig. 1), damage induction (Fig. 2) and cytokine production (Fig. 3), we hypothesized that Als3 may be the target on *C. albicans* hyphae that mediates discrimination between the yeast and hyphal form. Therefore, we screened the ability of the Δ*als* mutants to induce MKP1 phosphorylation or c-Fos production. Because 2 h was the time point previously shown to be optimal for characterization of the discriminatory response [16], it was used for this assay. MKP1 phosphorylation and c-Fos production were induced by the Δ*als* strains to a similar degree as by wild-type/parent cells (Fig. 4). This result indicated that deletion of individual *ALS* genes did not affect activation of epithelial cells via MPK1 or c-Fos and that Als3 was unlikely to be the hyphal moiety targeted by epithelial cells to discriminate between the yeast and hyphal form of *C. albicans*. Given that the Δ*als3* mutant was markedly reduced in its ability to cause epithelial damage (Fig. 2), the data also suggested that MKP1 or c-Fos activation was, in part, not directly correlated to damage as indicated previously [16].

### Increasing the fungal burden of Δ*als3* rescues damage and cytokine phenotypes

Our previous work demonstrated that epithelial activation and cytokine production in TR146 cells is dependent on fungal burdens [16]. Because the Δ*als3* mutant exhibited poor epithelial adherence, we hypothesized that this phenotype may account for the poor ability of Δ*als3* to induce damage and a cytokine response, since damage and cytokine induction require sustained hypha formation and epithelial activation [13], [17], [18]. Thus, it was possible that the deficiencies in the epithelial damage and cytokine responses for the Δ*als3* strain may be because the threshold level of activation was not reached due to insufficient numbers of Δ*als3* cells adhering to and invading the epithelial cells.

**Figure 5:**
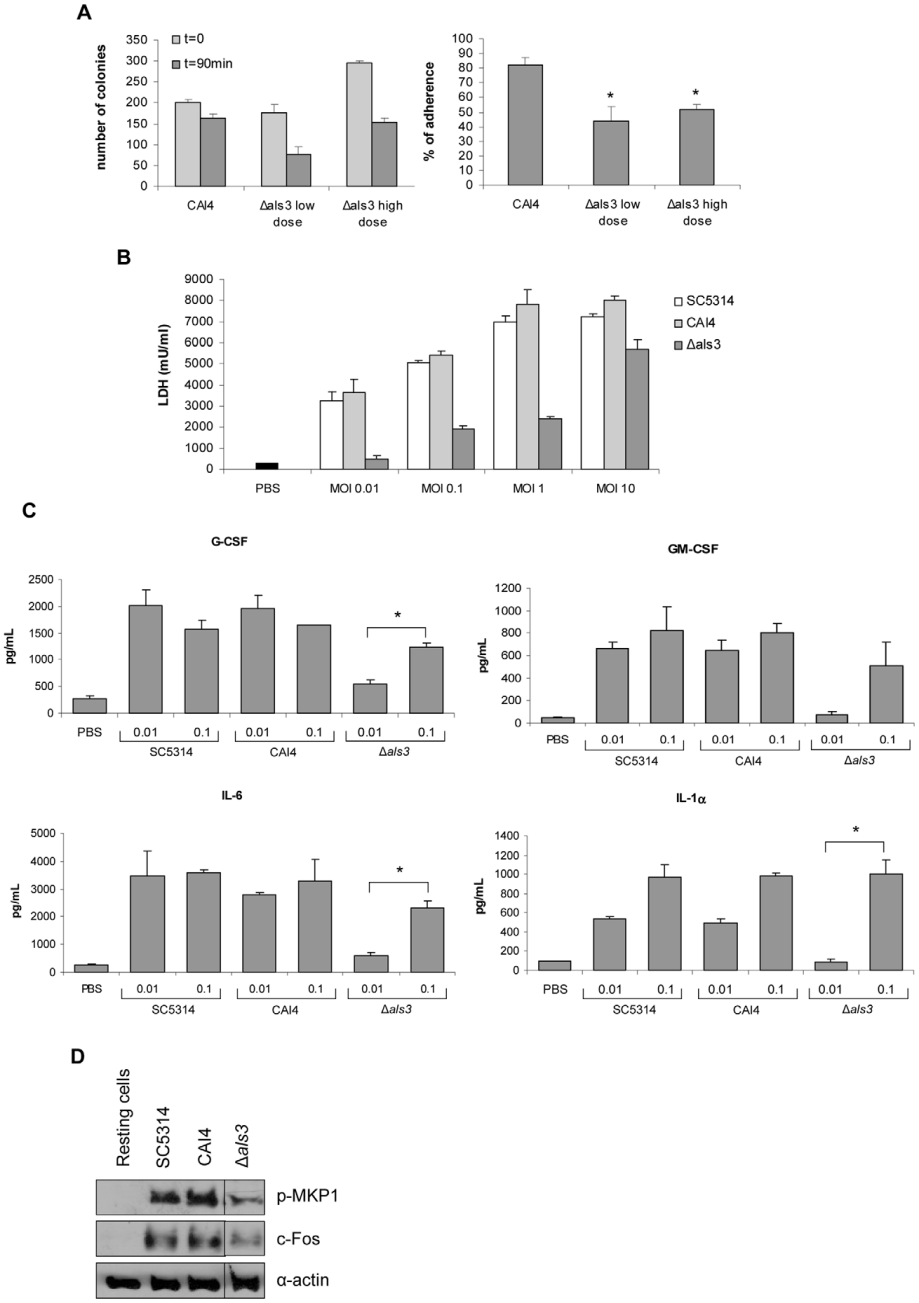
Adhesion, cell damage, cytokine production and activation of MKP1 and c-Fos by *C. albicans* Δ*als3* at different MOIs. *C. albicans* CAI4 yeast cells (200 cfu) and *C. albicans* Δ*als3*mutant yeast cells (200 cfu - low dose or 300 cfu - high dose) were added to TR146 monolayers and incubated for 90 min to determine the number of colonies and the percentage of adherence (A). *C. albicans* Δ*als3* mutant, the parent strain CAI4 and wild-type strain SC5314 were added to TR146 oral epithelial cells under standard culture conditions for 2 h or 24 h at a fungal:epithelial cell MOI ranging between 0.01 and 10. Induction of cell damage assessed by LDH release (B) and the production of cytokines by multiplex microbead assay (luminex) (C) were assayed in the cell culture medium supernatants at 24 h. (D) Induction of MKP1 phosphorylation and c-Fos at 2 h (MOI of 1). Data represent mean values ± SEM and are representative of duplicate experiments. * *p*<0.05 compared to CAI4.

First, we tested whether the percentage of adherence was increased if higher initial doses of Δ*als3* yeast cells were used. Although we did not observe an increase in the percentage of adherent cells by increasing the MOI, a greater number of Δ*als3* cells adhere to the epithelial cells as a result (Fig. 5a). The Δ*als3* shows a 50% reduction in adherence compared with the wild type strain CAI4. By increasing the number of Δ*als3* cells by 50% (from ∼200 (low dose) to ∼300 (high dose)) we observed a similar amount of adhered cells as with the parent strain when 50% fewer cells are used. This demonstrates that while the percentage of adherent cells does not change, more cells adhere if the number of cells is increased.

Next, we attempted to rescue the damage and cytokine phenotypes by increasing the Δ*als3* dose to the threshold activation level. We first inoculated TR146 monolayers for 24 h with SC5314, CAI4 and Δ*als3* at a MOI ranging from 0.01 to 10. Increasing the Δ*als3* burden 10-fold (from MOI of 0.01 to 0.1) was sufficient to initiate cell damage, albeit at a lower level than the wild-type/control strains (Fig. 5b). However, at a MOI of 10, Δ*als3* induced damage at similar levels as the wild-type/control strains, potentially indicating that a similar number of Δ*als3* cells were adhering and invading epithelial cells as the wild-type/control strains at an MOI of 0.1. The importance of fungal burdens was further supported by the observation that the LDH levels caused by SC5314 and CAI4 peaked and reached a plateau at a MOI of 1. Presumably, most or all TR146 cells were degraded at 24 h using this MOI, so increasing the MOI to 10 could not further increase epithelial damage. The Δ*als3* strain continued to increase LDH levels to a MOI of 10, consistent with the observation that the strain adhered poorly and required a greater fungal cell number to cause epithelial damage.

Next, we investigated whether the increased fungal burden could induce epithelial cytokine production by infecting TR146 cells for 24 h with *C. albicans* SC5314, CAI4 or Δ*als3* using MOIs of 0.01 and 0.1. Similar to the cell damage results, increasing fungal burden 10-fold was sufficient to induce cytokine production (Fig. 5c). For IL-1α, the increased MOI of the Δ*als3* strain produced cytokine levels similar to the control strains. Since IL-1α is associated with cell damage [31], [32], this result was consistent with the data that demonstrated increases in LDH when the Δ*als3* strain was used at a MOI of 0.1 (Fig. 5b). Together, the data suggested that the lack of damage and cytokine production observed for Δ*als3* at 24 h at lower MOIs was most likely due to its poor ability to adhere to and invade oral epithelial cells. Increasing the MOI restores all phenotypes by ensuring sufficient numbers of Δ*als3* cells adhere thus reaching the threshold level for activation. This also further demonstrates that a threshold level is required for epithelial cell activation (supporting our previous work [16], which is dependent on how many cells initially adhere. Furthermore, because cell damage and cytokine production were restored by increasing Δ*als3* burden, it was unlikely that Als3 was a hyphal factor that directly induced epithelial damage and cytokine production.

Although Als3 was unlikely to be the hyphal factor that directly activates epithelial cells, we hypothesized that *C. albicans* hyphal adhesion/anchoring to epithelial cells was a prerequisite for epithelial activation. In other words, sufficient numbers of hyphae first need to adhere to epithelial cells to enable the hyphal factor that activates MKP1/c-Fos to be present in sufficient quantity. Because we previously determined the threshold level of MKP1-C-Fos activation by wild-type *C. albicans* (SC5314) to be around a MOI of 1 [16] and that Δ*als3* adheres poorly to oral epithelial cells (Fig. 1), we reduced the Δ*als3* MOI from 10 to 1 with the prediction that MKP1/c-Fos activation would be reduced. As predicted, *C. albicans* Δ*als3* at a MOI of 1 only minimally induced MKP1 phosphorylation and c-Fos (Fig. 5d), further supporting the importance of fungal burdens and threshold levels of activation in *C. albicans* infection.

### Recombinant NT-Als3 does not induce epithelial damage, cytokines, MKP1 phosphorylation or c-Fos production

**Figure 6:**
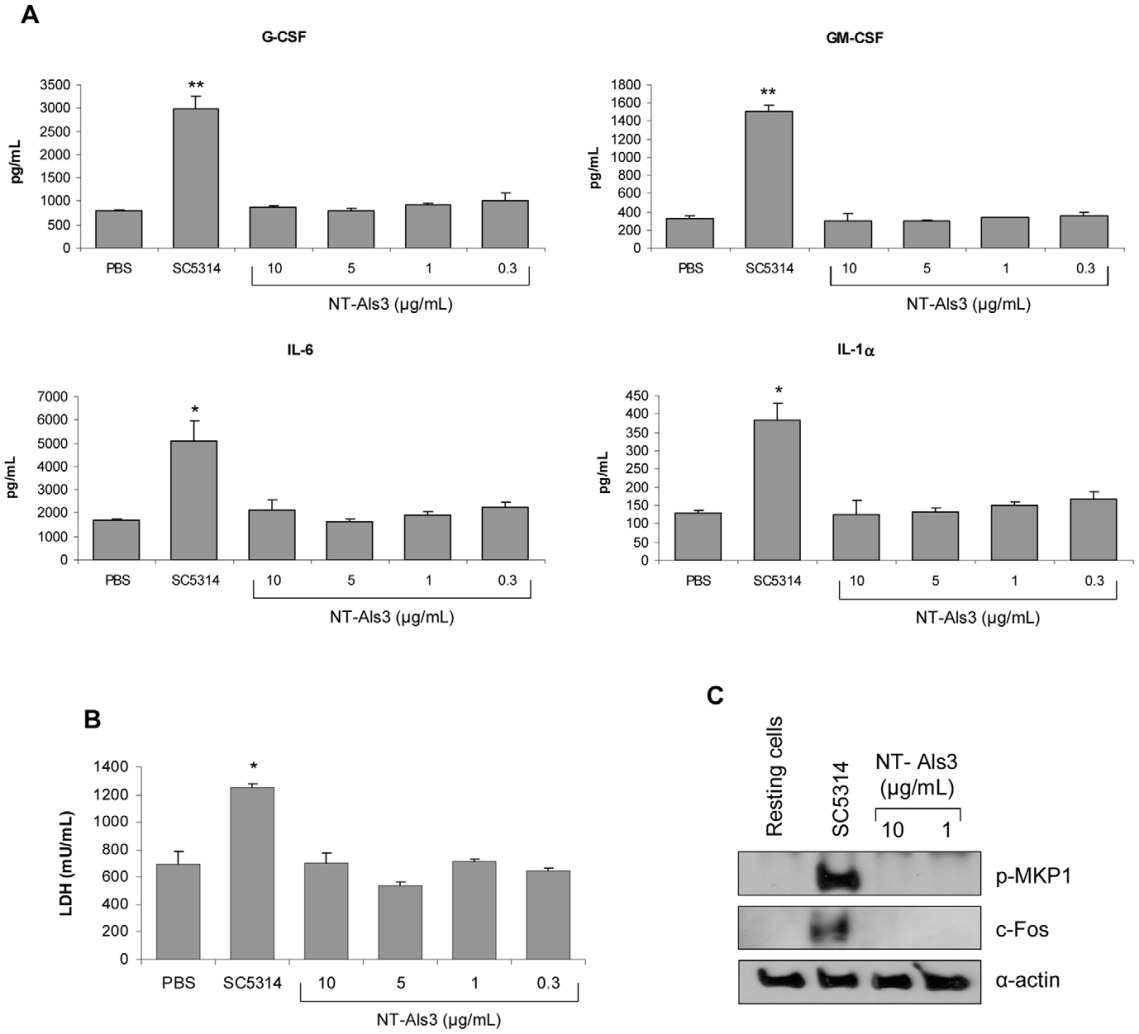
Stimulation of oral epithelial cells with NT-Als3 recombinant protein. Different concentrations of NT-Als3 ranging from 0.3–10 µg/ml and wild-type strain SC5314 were applied to TR146 oral epithelial cells and incubated at 37°C in 5% CO_2_ for 24 h (A, B) or 2 h (C) as described in Materials and Methods. Cell culture medium was collected and assessed for production of cytokines (A) and LDH release (B). (C) Total protein was isolated and induction of MKP1 phosphorylation and c-Fos assessed. Bands are shown relative to α-actin loading control. SC5314 was used in all assays at a fungal:epithelial cell MOI of 0.01 (A, B) or 10 (C). Data represent mean values ± SEM and are representative of duplicate experiments. * *p*<0.05; ** *p*<0.01 compared to SC5314.

The phenotypes observed for the Δ*als3* mutant suggested that Als3 might be required for initial epithelial adhesion, but not for direct induction of cell damage, cytokines or activation of the epithelial hypha discriminatory response. To test these ideas further, we stimulated TR146 epithelial monolayers with a purified recombinant portion of the Als3 protein [12]. This protein, called NT-Als3, includes amino acids 18 to 329 of the Als3 molecule and has an adhesive function [10]. We predicted that NT-Als3 would not induce cell damage, cytokine production, MKP1 phosphorylation or c-Fos because (i) Δ*als3* activated both MKP1 phosphorylation and c-Fos similar to the wild-type control at standard MOIs (Fig. 4) and (ii) damage and cytokine production were restored by increasing the Δ*als3* fungal burden (Fig. 5). NT-Als3 did not induce cytokine production (Fig. 6a), cell damage (Fig. 6b), MKP1 phosphorylation or c-Fos activation (Fig. 6c) despite testing multiple concentrations of protein. These data supported the conclusion that the N-terminal adhesive domain of Als3 was not responsible for directly activating immune responses or hyphal discrimination in oral epithelial cells.

## Discussion

The goal of this work was to investigate the role of Als proteins in epithelial adhesion and damage, and cytokine production. We also evaluated whether Als3 is the moiety that mediates activation of the MAPK-based MKP1/c-Fos signaling pathway. We found that Als3 makes an important contribution to *C. albicans* adhesion to TR146 oral epithelial cells, and subsequent epithelial damage, and that loss of Als3 results in reduced capacity of *C. albicans* to induce epithelial cytokines. The reduction in epithelial damage and cytokine production observed for the Δ*als3* strain was rescued by increasing the fungal burden in association with the epithelial cells. We found that Δ*als3* cells are still able to induce MKP1 phosphorylation and c-Fos production, indicating Als3 is not the target on *C. albicans* hyphae that mediates discrimination between the yeast and hyphal form. We also found that the soluble NT domain of Als3 was not sufficient to induce epithelial damage, cytokine production, MKP1 phosphorylation or c-Fos production.

The ability of Δ*als* strains to adhere to TR146 cells was studied previously in a different context. TR146 cells are the same as those used to produce recombinant human oral epithelium (RHE), marketed by SkinEthic (www.skinethic.com). The interaction between individual Δ*als* strains and oral RHE was described in previous publications [21]–[24]. The Δ*als3* strain was noted to have markedly reduced adhesion relative to the control strain. This assessment was made microscopically by counting *C. albicans* cells adhered to thick sections of the RHE. Using this analysis, the Δ*als2/P_Mal_-ALS2* strain also showed decreased adhesion to TR146, compared with the control. Microscopic inspection of thick sections also showed decreased RHE destruction by the Δ*als3* strain, and also by the Δ*als1* strain at 8 h post-inoculation.

Other assays have drawn conclusions about adhesion of Δ*als* mutants to epithelial cells. Using freshly collected buccal epithelial cells (BECs), the Δ*als3* strain showed significantly reduced adhesion relative to the control. Unexpectedly, the Δ*als5*, Δ*als6*, and Δ*als7* strains showed nearly a two-fold increase in adhesion to BECs. Phan et al. [33] used a differential fluorescence assay to identify association between *C. albicans* and epithelial cells from the FaDu pharyngeal carcinoma cell line. They concluded that Als3 is not involved in adhesion to epithelial cells, but is involved in invasion. In the adhesion assays described here, only the Δ*als3* strain showed a significant decrease in adhesion, compared with control strains. Overall, a variety of approaches has been used to address the role of Als proteins in interactions between *C. albicans* and epithelial cells, with some variability in the conclusions. Undoubtedly, the differing assay configurations, such as whether yeast cells or germ tubes are inoculated into the assay, assay timing, and the fungal burden used for each study, contribute to these differences. Regardless of the differences, the conclusion that Als3 plays a greater role in epithelial cell interactions than the remainder of the Als proteins, is consistent across the body of evidence, and our results further support this conclusion.

These data raise the question of why Als3 has a greater role in epithelial interactions between *C. albicans* germ tubes and epithelial cells than the other Als proteins. Among the possible explanations are relative Als protein abundance and spatial distribution on the germ tube, and functional interchangeability between the various Als proteins. Studies of *ALS* gene expression provided the first clues to the possible relative abundance of Als proteins (reviewed in [7]). Some *ALS* genes are regulated by large increases and decreases in transcriptional level, while others are transcribed consistently at lower levels. *ALS3* has a large increase in transcription when *C. albicans* yeast cells are placed into growth conditions that promote hypha formation [34]. Analysis using Als3-specific monoclonal antibodies visualized the great abundance and broad distribution of Als3 over the entire surface of *C. albicans* hyphae [12]. This abundance and distribution promote the greatest opportunity for contact with epithelial cells, potentially resulting in the greatest phenotypic effect on adhesion.

Previously, a potential role for Als1 and Als5 in epithelial adhesion was suggested [35], which seems inconsistent with our results that demonstrate lack of an adhesive change in *C. albicans* strains from which *ALS1* or *ALS5* were deleted. However, results in the earlier study were observed for *S. cerevisiae* strains in which each *ALS* gene was overexpressed. The overexpression strains have a uniform, thick coat of Als protein, unlike the wild-type abundance and distribution of the Als1 and Als5 on wild-type *C. albicans* [36], [37]. Understanding the differences in protein presence in each experimental approach provides consistent conclusions from seemingly disparate results: Als1 or Als5 can mediate epithelial cell adhesive interactions, given a sufficient abundance on the cell surface. These observations further solidify our conclusion that the greatest phenotypic effect is observed for Als3, because of its abundance and broad coverage of *C. albicans* hyphae. Experiments to address the functional interchangeability of Als proteins have not been fully explored.

Since adhesion is a prerequisite for invasion and Als3 is also an invasin, the reduced ability of Δ*als3* to adhere is sufficient to account for its significantly reduced ability to cause subsequent cell damage. Because the Δ*als3* mutant is able to form normal germ tubes and hyphae, its inability to adhere and invade epithelial cells most likely explains why the Δ*als3* mutant was also unable to induce cytokines, since cytokine induction requires sustained adhesion and invasion by hyphae [13], [17], [18]. This conclusion is supported by data showing that induction of epithelial damage and cytokine production could be restored by simply increasing the fungal burden of Δ*als3* 10-fold (from MOI of 0.01 to 0.1). Therefore, our data support the notion that a threshold level first needs to be reached to fully activate epithelial cells and that this depends on how many cells have initially adhered. Notably, the lack of invasion by Δ*als3* at lower MOIs (0.01) also correlates with the lack of IL-1α production (a cytokine associated with cell damage), which again could be restored by increasing the fungal burden of Δ*als3* 10-fold (to MOI of 0.1).

Recently, we found that epithelial cells are strongly activated by *C. albicans* hyphae via a MAPK-based mechanism constituting MKP1 phosphorylation and c-Fos activation, contributing to cytokine production and associated with cell damage [16] thus initiating a ‘danger response’. It is known that *C. albicans* entry into epithelial cells via induced endocytosis is mediated via E-cadherin through a mechanism that is actin-dependent and requiring clathrin [38]. Therefore, although the predominant function of Als3 is to mediate epithelial adherence and entry, we hypothesized that once inside the epithelial cells Als3 may trigger the epithelial cell danger response mechanism (specifically MKP1 phosphorylation and c-Fos production), thus enabling epithelial cells to discriminate between the yeast and hyphal form. However, the Δ*als3* mutant was able to induce MKP1 phosphorylation and c-Fos equally as well as wild-type/parent cells whilst NT-Als3 was unable to directly induce MKP1 phosphorylation or c-Fos. These data indicate that Als3 does not activate the yeast-hyphal discriminatory signaling response in oral epithelial cells and that its role is most likely confined to promoting *C. albicans* adhesion and to mediating initial invasion of *C. albicans* via induced endocytosis. However, we note that these conclusions are based in part on NT-Als3 (albeit supported by the Δ*als3* data), so we cannot exclude that other regions of the Als3 protein may be involved in epithelial cell activation through other indirect mechanisms. Additional investigations are underway to identify the *C. albicans* hypha factor that mediates activation of the MAPK-based MKP1/c-Fos signaling pathway.
